# Hypoxic-ischemic related cerebrovascular changes and potential therapeutic strategies in the neonatal brain

**DOI:** 10.1002/jnr.24590

**Published:** 2020-02-14

**Authors:** Clémence DISDIER, Barbara S STONESTREET

**Affiliations:** 1 Department of Pediatrics, Women & Infants Hospital of Rhode Island, The Alpert Medical School of Brown University, Providence, RI 02905, USA

**Keywords:** neurovascular unit, hypoxia ischemia, brain injury, neonates

## Abstract

Perinatal hypoxic-ischemic (HI)-related brain injury is an important cause of morbidity and long-standing disability in newborns. The only currently approved therapeutic strategy available to reduce brain injury in the newborn is hypothermia. Therapeutic hypothermia can only be used to treat HI encephalopathy in full term infants and survivors remain at high risk for a wide spectrum of neurodevelopmental abnormalities as a result of residual brain injury. Therefore, there is an urgent need for adjunctive therapeutic strategies. Inflammation and neurovascular damage are important factors that contribute to the pathophysiology of HI-related brain injury and represent exciting potential targets for therapeutic intervention. In this review, we address the role of each component of the neurovascular unit (NVU) in the pathophysiology of HI-related injury in the neonatal brain. Disruption of the blood-brain barrier (BBB) observed in the early hours after an HI-related event is associated with a response at the basal lamina level, which comprises astrocytes, pericytes, and immune cells, all of which could affect BBB function to further exacerbate parenchymal injury. Future research is required to determine potential drugs that could prevent or attenuate neurovascular damage and/or augment repair. However, some studies have reported beneficial effects of hypothermia, erythropoietin, stem cell therapy, anti-cytokine therapy and metformin in ameliorating several different facets of damage to the NVU after HI-related brain injury in the perinatal period.

## 1 | Introduction

The Blood-Brain barrier (BBB) is an important contributor to the maintenance of central nervous system (CNS) homeostasis that prevents the entry of many detrimental substances into the nervous tissue. The brain endothelial cells (BECs) of the BBB supply nutrients and other essential molecules from blood to brain cells and eliminate waste products to provide an optimal environment for brain development. The BECs are also important for signal transduction including numerous immune responses. For example, immune blood born cell trafficking is regulated in part by the expression of adhesion molecules on BECs. It is important to emphasize that BECs by themselves are not capable of intrinsically forming a fully functional BBB. The relatively new concept of neurovascular unit (NVU) underscores the requirement for coordinated cell interactions that include BECs, the basal lamina, the surrounding glial limitans, pericytes, microglia and neurons (Hawkins & Davis, 2005; Iadecola, 2017). The function of this unique barrier requires interactions and coordination among the multiple cellular constituents and the extracellular matrix (ECM) to establish an effective anatomical and functional barrier system.

BECs possess the most characteristic features of the barrier in the brain, which include reduced paracellular transport as a result of tight junctions (TJ) between neighboring BECs and the expression of efflux and influx transporters that regulate the brain microenvironment (Ek, Dziegielewska, Habgood, & Saunders, 2012; Saunders, Liddelow, & Dziegielewska, 2012; Strazielle & Ghersi-Egea, 2015; Virgintino et al., 2000). TJs are present and functional early in brain development both in laboratory animals (Bauer et al., 1993; Daneman, Zhou, Agalliu, et al., 2010; Ek, Dziegielewska, Stolp, & Saunders, 2006; Ek, Habgood, Dziegielewska, & Saunders, 2003; Hirase et al., 1997; Kniesel, Risau, & Wolburg, 1996) and in human fetuses (Anstrom, Thore, Moody, & Brown, 2007; Ballabh, Hu, Kumarasiri, Braun, & Nedergaard, 2005; Virgintino et al., 2004). The vascular basal lamina components appear associated with detectable occludin and claudin-5 in the primary cerebral cortical vessels by 12 weeks of the human gestation (Anstrom et al., 2007; Ballabh et al., 2005; Virgintino et al., 2004; Virgintino et al., 2007). Claudins and occludin reorganize to concentrate in linear discontinuous tracts to form immature TJ strands between week 12 and 14 weeks of the human gestation (Virgintino et al., 2004). TJ proteins display the adult like appearances at the junctional area between endothelial cells a few weeks later. The physical barrier mechanism(s) established early in development facilitate a stable environment needed for brain maturation. On the other hand, the ontogeny of transporters at the BBB show that the expression of some carriers differ between the neonatal and the adult brain (Ek, D’Angelo, Lehner, et al., 2015; Ek et al., 2012; Ek et al., 2010; Harati, Benech, Villegier, & Mabondzo, 2013; Lam et al., 2015; Mollgard, Dziegielewska, Holst, Habgood, & Saunders, 2017; Soares, Do, Mabondzo, Pons, & Chhun, 2016). The developmental expression shows different profiles depending on transporters in both humans and animals. The NVU is also a metabolic barrier because BECs and other NVU cells express metabolic enzymes for neurotransmitters and cytochromes P450 (Decleves et al., 2011; Shawahna et al., 2011). The ontogeny of all these systems has not yet been fully delineated in the developing brain. Developmental changes in the expression of pumps, transporters and metabolic enzymes may reflect specific requirements of a developing brain and, therefore, the effects of injury during development could differ from those in the adult brain.

A number of brain related injuries observed in neonates could also be caused by genetic-related and other factors that occur during antenatal brain development (MacLennan, Thompson, & Gecz, 2015). Sometimes the etiological basis of adverse neurological and behavioral symptoms cannot be determined by the clinical events occurring during the perinatal period. Despite these factors and in contrast to the “mechanistic” approach provided by MacLennan et al., other etiologies of neonatal brain injury potentially include perinatal stroke, intraventricular hemorrhage and asphyxia with hypoxia/ischemia, which could have similar pathways predisposing to injury (Hagberg et al., 2015). The main molecular pathways activated by the simultaneous actions of HI and genetic abnormalities could be associated with the subsequent HI-related perinatal brain injury. However, these factors remain controversial because the protective effects of mild anoxia/hypoxia have also been demonstrated in animal models of perinatal brain injury (Alotaibi, Arrowsmith, & Wray, 2015).

Normal brain development is highly dependent on a sufficient blood, oxygen and energy supply. HI-related brain injury is a result of an insufficient blood flow to the brain combined with lower-than-normal concentrations of oxygen in the arterial blood. These events in the immature and developing brain cause significant mortality and can result in long-term neurological deficits such as cerebral palsy, epilepsy and seizure disorders, developmental delay, severe learning disabilities, cognitive, motor and behavioral abnormalities (Conklin, Salorio, & Slomine, 2008; Fatemi, Wilson, & Johnston, 2009; Kharoshankaya et al., 2016; Pappas et al., 2015). Randomized controlled trials of therapeutic hypothermia in full term infants have demonstrated a decrease in death or severe disability 18 months after the insult. However, death and disabilities continue to occur in 30 to 70% of infants regardless of treatment with therapeutic hypothermia (Azzopardi et al., 2010; Shankaran et al., 2005; Tagin, Woolcott, Vincer, Whyte, & Stinson, 2012). After the relative success of translating therapeutic hypothermia from the bench to the bedside for neuroprotection, adjunctive treatments to further enhance the neuroprotective efficacy of hypothermia have recently been extensively investigated. HI-related brain injury is characterized by a pronounced neurovascular inflammatory response (Back, 2006; Bartha et al., 2004; Hagberg et al., 2015; Liu & McCullough, 2013) along with early structural vasculature alterations (Moretti et al., 2015). Both inflammation and BBB abnormalities can contribute to neuronal damage. Therefore, neurovascular inflammation and vasculature structural alterations represent basic mechanisms in HI-related brain injury and, consequently, potential therapeutic targets. Elucidating the mechanisms underlying the responses of the NVU to HI in the neonatal brain is critical because they could give insights into potential endogenous neuroprotective pathways that could suggest therapeutic targets. The purpose of this review is to summarize the current knowledge regarding effects of HI-related injury to the NVU in the neonatal brain. In addition, we also summarize the effects of several therapeutic interventions such as erythropoietin or stem cell therapy, which are the only therapies that have been examined with reference to changes in the NVU.

## 2 | The neonatal neurovascular unit in hypoxic ischemic conditions

### 2.1 | From HI animal models to human NVU

Animal models used for the study of HI-related brain injury in neonates have been reviewed in detail by others (Ashwal & Pearce, 2001; Hagberg, Ichord, Palmer, Yager, & Vannucci, 2002; Koehler, Yang, Lee, & Martin, 2018; Millar, Shi, Hoerder-Suabedissen, & Molnar, 2017; Rumajogee, Bregman, Miller, Yager, & Fehlings, 2016). These reviews have addressed interspecies differences as well as the appropriateness of various animal models including rodents to study HI-related brain injury. The characteristics of brain development have been established in several species to support the use of appropriate animal models to study neonatal brain injury (Clancy, Darlington, & Finlay, 2001; Clancy, Finlay, Darlington, & Arland, 2007; Dobbing & Sands, 1979). The most widely used model of HI-related brain injury is the Rice-Vannucci model, which combines unilateral carotid artery ligation with exposure to variable lengths of hypoxia in 7-day-old rat pups (Rice, Vannucci, & Brierley, 1981).

There is now sufficient evidence to justify the use of 7-days old rats because the brain maturation is thought to be roughly comparable to that of the third trimester human fetus (Clancy et al., 2001). Nonetheless, various species have been categorized into prenatal, perinatal and postnatal brain developers with reference to their brain growth velocity relative to the time of birth (Dobbing & Sands, 1979). In this regard, the human and pig exhibits mainly perinatal brain growth, whereas the rodent brain matures after birth (Dobbing & Sands, 1979). Studies have revealed that the structure of the NVU exists and is relatively similar among a variety of species including rodents and large animals such as the piglet and lamb that have been used to study HI-related brain injury (O’Brown, Pfau, & Gu, 2018). The structure of the NVU in the human brain appears to be like that of other mammals. However, the NVU in the human brain exhibits several unique features including a greater number of astrocytes with a more elaborate astrocytic network than observed in the rodent brain (Oberheim, Wang, Goldman, & Nedergaard, 2006). The species differences in the features of the NVU have not been investigated in the developing brain with reference to the complex molecular interactions among the cellular elements (brain endothelial cells, astrocytes, pericytes, inflammatory cells, and neurons) and the acellular (basal lamina) components of the NVU that cooperatively contribute to BBB integrity. Therefore, the translation of observations from HI-related brain injury in animal models to the developing human neonate needs to be considered with caution.

### 2.2 | Endothelial cells

BECs represent the fundamental cell type of the BBB because they are the first cells to encounter substances in the systemic circulation. The BECs differ considerably from classic endothelial cells in peripheral vessels because they contain a large quantity of cytosolic mitochondria, low pinocytotic activity and lack fenestrations. The tight junctions (TJ) between neighboring cells form the physical barrier separating blood stream and brain parenchyma and resulting in very limited paracellular passage. Various transport systems are present on abluminal and luminal membranes also regulate the transcellular traffic. These polarized transporters are responsible for the selective ‘transport barrier’ in order to meet the high nutritional and energy requirements of the brain and to exclude potentially harmful substances such as exogenous toxins circulating in blood.

#### 2.2.1 | HI related changes in permeability

Disturbances in endothelial permeability have been evaluated by several methods and described in multiple models of HI-related injury in the fetal and neonatal brain (Table 1). Many of these studies were performed using the Rice-Vannucci model in rodents and describe extravagation of blood born molecules such as albumin or IgG within a few hours after the insult. Some studies also reported increases in permeability to small injected molecules such as sucrose, dextran or fluorescein. Normalization in barrier function has been observed to range from 3 to 7 days after barrier opening resulting from HI-related events. The differences observed in the time of recovery may be a result of differences in the models used and the extent of brain injury. The effects of ischemic-reperfusion related brain injury and umbilical cord occlusion on BBB function has been examined in fetal sheep. Several studies have demonstrated similar increases in BBB permeability. In summary, there is agreement that the HI and/or ischemic injury in the neonatal or fetal brain induce an increase in BBB permeability.

Findings in human neonates appear to be consistent with these observations because albumin concentrations in cerebro-spinal fluid compared with plasma levels are increased in infants that had been exposed to hypoxic-ischemic encephalopathy (HIE) (Aly et al., 2009; Kumar, Mittal, Khanna, & Basu, 2008). The impaired supply of oxygen and glucose after HI-related injury results in an anaerobic stress and initiates a cascade of ATP depletion, efflux of excitotoxic glutamate, ion shifts, and metabolic imbalances associated with acidosis, oxidative stress, and release of pro-inflammatory cytokines. These stress induced signals can directly affect BBB permeability, predispose to cerebral edema and promote entry of neurotoxic substances into the brain parenchyma (N. Joan Abbott & Friedman, 2012; Stanimirovic & Satoh, 2000). Endothelial activation and increases in BBB permeability contribute to HI-related brain injury by intensifying the exposure to pro-inflammatory mediators in the systemic circulation and by initiation and further propagation of the inflammatory waves across the brain parenchyma. Mechanisms underlying these changes have largely been described in the adult brain (Kaur & Ling, 2008; Yang, Hawkins, Dore, & Candelario-Jalil).

The tightness of the BBB endothelium is a result of TJ proteins that seal neighboring BECs and restrict the diffusion from the lumen of the vessel into the brain parenchyma. The TJs are composed of a combination of transmembrane (claudins family and occludin) and cytoplasmic (i.e. zonula occludens (ZO) family) proteins that interact with the actin cytoskeleton. Decreases in the expression of TJ proteins have been reported after HI-related injury in the rodent brain (W. Q. Chen et al., 2009; Ek, D’Angelo, Baburamani, et al., 2015; Fang et al., 2017; Ma, Dasgupta, Li, Huang, & Zhang, 2017; W. T. Zhang et al., 2016) and after ischemia-reperfusion in the brain of fetal sheep (Malaeb, Sadowska, & Stonestreet, 2007; Sadowska et al., 2012) in association with increases BBB permeability. The ontogeny of TJs and the measure of BBB integrity in the developing brain demonstrated that the barrier is fully functional from early in CNS development (Daneman, Zhou, Agalliu, et al., 2010; Ek et al., 2006; Ek et al., 2003), therefore, it is not surprising that effect of HI on alterations permeability are somewhat similar between the adult and neonatal brain vasculature. However, whether the neonatal BBB can recover after this initial increase of permeability remains to be determined. Alterations in BBB permeability are often observed in epilepsy long after an exposure to HI-related brain injury (Bankstahl et al., 2018; Marchi, Granata, Ghosh, & Janigro, 2012; van Vliet et al., 2007). Thus, further investigation is required to fully understand the recovery of the barrier properties and long-term consequences to the immature brain. The developmental status of the cerebral vasculature could be a critical contributing factor to the pathogenesis of HI-related brain injury. Muramatsu et al. showed that the BBB of younger rat (P7) was more vulnerable to HI damage compared with older rats (P21) (Muramatsu, Fukuda, Togari, Wada, & Nishino, 1997). Moreover, most of the studies summarized above studied BBB function in animal models that simulated the cerebral development similar to the brain of full-term human infant. Studies have not yet evaluated the effects of HI insults on BBB permeability in animal models that are similar to the premature human brain.

In addition, there is increasing evidence of sexual dimorphism in response to neonatal HI-related injury that remains to be explained (Charriaut-Marlangue, Besson, & Baud, 2018). Immune related modifications in the BBB have been shown to be sex-dependent in the adult (Erickson et al., 2018). However, the sex related differences in BBB permeability has not been examined in the neonatal brain.

#### 2.2.2 | Transport system dysfunction

The transport systems at the BBB have two main purposes: To carry essential molecules (nutrients, amino acids, etc.) and to limit the entry of xenobiotics into the brain via the efflux system. In this regard, the ATP binding cassette (ABC) transporters P-glycoprotein (Pgp) (Lam et al., 2015), Breast Cancer Resistance Protein (BCRP) (Eisenblatter, Huwel, & Galla, 2003), Multidrug Resistance-associated Proteins (MRPs) MRP4 and MRP5 (Seetharaman, Barrand, Maskell, & Scheper, 1998), and OATP1A2 and OATP2B1 along with the Organic Anion Transporting Polypeptides (OATPs) (Roth, Obaidat, & Hagenbuch, 2012) are considered the most clinically significant transporters within the BECs. The ontogeny of transporters at the BBB show that the expression of some carriers differ between the neonatal and adult brain (Ek, D’Angelo, Lehner, et al., 2015; Ek et al., 2012; Ek et al., 2010; Harati et al., 2013; Lam et al., 2015; Mollgard et al., 2017; Soares et al., 2016; Strazielle & Ghersi-Egea, 2015; Tsai et al., 2002). The activity of transporters at the molecular level can be modulated by a numerous physiological signals such as activation of intracellular protein kinases and transcription factors (Miller, 2015). HI results in the release of an array of complex signaling molecules that can enhance these signaling pathways including, reactive oxygen species, pro-inflammatory cytokines and chemokines.

Radiolabeled interleukin-1 β (IL-1 β) transfer across the BBB has been shown to be increased at 4 h and 24 h after exposure to ischemic brain injury in fetal sheep *in utero* (Patra et al., 2017; Sadowska et al., 2012). The increases in IL-1 β transfer were most likely explained by changes in the activity and/or expression of a specific IL-1 transporter at the BBB. Consistent with this finding, previous work has shown that there is a proportionately greater propensity for cytokines to cross the BBB despite their large size as a result of saturable transport systems (William A. Banks, 2010; W. A. Banks, Kastin, & Broadwell, 1995; Threlkeld et al., 2010). Even though the specific carrier for IL-1 β has not been identified yet, these observations suggest that HI could trigger neurovascular inflammatory responses that could modify BBB transport systems.

Glucose, an essential substrate for brain oxidative metabolism, is transported across the BBB into glia and neurons by a family of structurally related membrane spanning glycoproteins called facilitative glucose transporters (glut) (Vannucci & Vannucci, 2000). Glucose transporters belong to the solute carrier (SLC) transporter family. Fung et al demonstrated that brain injury was augmented in glut 3 deficient mice after exposure to an HI insult (Fung et al., 2010). This study emphasizes the importance of the glut 3 transporter in neonatal HI-related brain injury. However, modifications in the activity and/or expression of transporters critical to the supply of metabolic substrates have not been investigated in wild-type rodents after HI-related injury in the neonatal brain.

The expression of major efflux and influx pumps, such as the ABC or SLC transporters, have not been investigated in the neonatal brain after HI injury, even though they have a major role in BBB function. However, studies in adults suggest that ischemic stroke can modify BBB transporter function. For example, P-glycoprotein (P-gp/ MDR1/ ABCB1), a particularly important ABC transporter that is responsible for handling a wide variety of endogenous substrates and xenobiotics, is upregulated after ischemic stroke in rodents (Cen et al., 2013; Cui et al., 2015; Spudich et al., 2006). The breast cancer resistant protein (BCRP/ABCG2) expression is also increased in the peri-infarct areas (Dazert et al., 2006; Shin et al., 2018).

In summary, there is a paucity of information regarding the regulation of transport activity across the BBB after HI-related injury in the neonatal brain. It is likely that there is an age dependent response of the transporters to HI in the neonatal brain because ontogenic modulations in transporters have been reported at the BBB during development. Transporters (i.e. ABC and SLC) provide a considerable opportunity to protect the BBB and/or promote BBB repair by facilitating endothelial uptake of drugs with cytoprotective/antioxidant properties or by preventing cellular loss of critical endogenous substances. However, further work is required to provide information regarding the interplay of cell-to-cell interactions, transporters, and signaling pathways at the BBB endothelium to understand how these systems could be effectively targeted. Furthermore, these systems are extremely important and require further investigation because infants with HIE are currently exposed to numerous medications for which these transporters could have important effects on brain drug concentrations and deposition.

#### 2.2.3 | Metabolism

BECs express several enzymes responsible for the metabolism of neurotransmitters. For example, they express monoamine estherases, cholinesterases, GABA transaminases, aminopeptidases and endopeptidases. BECs also express different systems responsible for the metabolism of drugs typically found in the liver, such as the cytochromes P450 (CYP450 or phase I enzymes) and phase II enzymes of metabolism (Daneman, Zhou, Agalliu, et al., 2010; Decleves et al., 2011; Munji et al., 2019; Shawahna et al., 2011). Toxin and drug-metabolizing enzymes contribute along with the efflux transporters to detoxification at the BBB (El-Bacha & Minn, 1999). In addition, enzymes are important to metabolize endogenous substrates such as fatty acids, hormones, steroids, and vitamins and regulate the concentration of signaling pathway molecules. The ontogeny of the different enzyme systems has not been examined in the developing brain or with respect to their regulation after injury. The effects of HI-related injury on metabolic enzymes and their regulation at the BBB have been investigated even in the adult brain.

HI-related brain damage is mediated through numerous overlapping mechanisms resulting in cell death: excitotoxicity, oxidative stress and inflammation (Back, 2015; Back & Miller, 2014; Ferriero, 2004; Gonzalez & Ferriero, 2008; Jantzie et al., 2015; Northington, Graham, & Martin, 2005). The mechanisms underlying the vulnerability of the immature brain to cell death remain controversial (Northington, Chavez-Valdez, & Martin, 2011; Northington et al., 2005). This sensitivity might be partly explained by the higher expression of the N-methyl-D-aspartate (NMDA) receptor in the developing brain compared to the adult. The NMDA receptor is an ionotropic glutamate receptor stimulated during excitotoxicity that is expressed at 150–200 percent higher amounts in P6 rats compared with those in adults (Tremblay, Roisin, Represa, Charriautmarlangue, & Benari, 1988). Apoptosis, necroptosis/necrosis as well as autophagic cell death are mechanisms described after HI-related brain injury in the immature brain. However, studies have not examined the specific sensitivity of the endothelium to cell death in context of HI-related brain injury in the neonatal brain.

#### 2.2.4 | The extracellular matrix, angiogenesis and neovascularization

The basal lamina membrane is composed of ECM proteins (collagen sub-units, heparin sulfate proteoglycans, laminin, fibronectin, etc.) (Ballabh, Braun, & Nedergaard, 2004; Hawkins & Davis, 2005; Zlokovic, 2008). BECs and pericytes, as described in detail below, are in direct contact with the basal lamina.

ECM glycoprotein staining can be used to examine vasculature density after HI injury in the neonatal brain. Laminin was used as maker to show decreased vessel density after umbilical cord occlusion fetal sheep. Decreases in vessel density was observed in the caudate nucleus along with shifts in the frequency of smaller to larger blood vessels in periventricular and subcortical white matter. In contrast, blood vessel density and morphology did not change after ischemia in the cerebral cortex (Baburamani, Lo, Castillo-Melendez, & Walker, 2013). Blood vessel density also decreased after HI-related injury in rodents (Hsu et al., 2014) and newborn piglets (Jain, Kratimenos, Koutroulis, Buddhavarapu, & Ara, 2017). Several studies have suggested that angiogenesis promotes neovascularization within several days after ischemic brain injury in neonatal rodents. These findings are based on results from a variety of models including neonatal stroke (D. Z. Mu et al., 2003) and prolonged hypoxia (Ogunshola et al., 2000). Morphometric analysis of cerebral cortical microvessels showed that neovascularization began at approximately 72 h after transient carotid occlusion in fetal sheep (Virgintino et al., 2014).

Vascular endothelial growth factor (VEGF) is a key mediator of vessel proliferation that is up regulated after neonatal HI. VEGF signaling pathways have been shown to stimulate angiogenesis after recovery from HI-related brain injury in neonatal rodents (Lyons, Anderson, & Meyer, 1991; Shimotake, Derugin, Wendland, Vexler, & Ferriero, 2010; Tang et al., 2017). Significant increases in the percentage of VEGF expressing blood vessels were observed in the subventricular zone and in periventricular and subcortical white matter 24 and 48 hours after umbilical cord occlusion in the ovine fetus (Baburamani, Castillo-Melendez, & Walker, 2013). Although VEGF up-regulation in support of neovascularization is potentially neuroprotective, the up-regulation also increases BBB permeability resulting in subsequent leakage of blood-born substances into the brain parenchyma and predisposition to brain edema (Baburamani, Ek, Walker, & Castillo-Melendez, 2012).

Basic fibroblast growth factor (FGF-2) and hypoxia-inducible factor-1 α (HIF-1 α) are two other growth factors that have been shown to augment angiogenesis after FI I in neonatal rodents (Lu, Jiang, Zhu, Zhang, & Wang, 2014; Lyons et al., 1991; D. Z. Mu et al., 2003; Tang et al., 2017). HIF-1α is *a* nuclear factor that modulates many Hl-related processes including neovascularization by regulating the transcription of its downstream elements such as VEGF. Increases in cerebral cortical FGF-2 was also associated with increases in neovascularization in the ovine cerebral cortex after ischemia (Virgintino et al., 2014).

Matrix metalloproteinases (MMPs) are a family of zinc endopeptidases. They are responsible for remodeling the extracellular matrix including the basal lamina of the BBB, regulating cell receptors and cleaving their ligand binding domains in the brain (Bonnans, Chou, & Werb, 2014; Kim, Turnbull, & Guimond, 2011; Nagase, Visse, & Murphy, 2006; Visse & Nagase, 2003). Tissue injury and inflammatory responses after HI insults can result in matrix degradation and predispose to disruption of the BBB. MMPs are mainly released from activated microglia and infiltrating leukocytes but they are also highly expressed in BECs and astrocyte end-feet processes. There is now substantial information demonstrating that degradation of basement membrane proteins by several MMPs that are elevated after ischemia and HI in the fetal and neonatal brain (W. Q. Chen et al., 2009; X. Chen, Patra, Sadowska, & Stonestreet, 2018; Leonardo et al., 2008; Ranasinghe et al., 2009; Savard et al., 2015; Svedin, Hagberg, Savman, Zhu, & Mallard, 2007; W. T. Zhang et al., 2016). Pro-inflammatory cytokines stimulate the proteolytic activity of MMPs. However, MMPs also enhance inflammatory responses by cleavage of pro-inflammatory cytokines attached to cell surfaces releasing these molecules into the extracellular milieu (Gearing et al., 1995; Schonbeck, Mach, & Libby, 1998).

### 2.3 | Pericytes

Pericytes surround the BBB providing additional separation of the blood components from neural tissue. They are evenly spaced along the walls of capillaries and share a common basal lamina with BECs. Pericytes are also termed vascular smooth muscle cells or myofibroblasts of blood vessels because of their ability to contract and, consequently, regulate local microvascular blood flow (Bandopadhyay et al., 2001; Cardoso, Brites, & Brito, 2010). They also have a critical role in cellular communication within the NVU. Gap junctions enable direct exchange of ions and small molecules between pericytes and BECs. In addition, they preserve the structure of the BBB by stimulating TJ formation (Ballabh et al., 2004; Daneman, Zhou, Kebede, & Barres, 2010; Dente, Steffes, Speyer, & Tyburski, 2001; Nakagawa et al., 2007). They also have a critical role in the development, maturation, remodeling of blood vessels (Paula Dore-Duffy & Cleary, 2011; Hirschi & Damore, 1996) and in inflammatory processes (Dalkara, Gursoy-Ozdemir, & Yemisci, 2011; Hawkins & Davis, 2005).

There is a paucity of information regarding the role of pericytes after HI in the neonatal brain. Most of the information regarding the response of pericytes to HI has been reported in the adult brain. Pericytes migrate away from microvessels after HI in the adult brain (P. Dore-Duffy et al., 2000; Duz, Oztas, Erginay, Erdogan, & Gonul, 2007; Fernandez-Klett et al., 2013; Gonul et al., 2002; Hall et al., 2014). Perivascular cells are not the first cells to respond to HI-related brain injury and are less sensitive than BECs to HI injury (Engelhardt, Huang, Patkar, Gassmann, & Ogunshola, 2015). They also regulate cellular interactions at the NVU and TJ protein localization in BECs *in vitro* during exposure to hypoxia (Al Ahmad, Taboada, Gassmann, & Ogunshola, 2011). Likewise, they reduce apoptosis in BECs thereby stabilizing the endothelium (Ramsauer, Krause, & Dermietzel, 2002). However, the potential beneficial effects of pericytes to preserve the structural integrity of the barrier remain to be examined *in vivo.* Several other observations also support concept that pericytes stimulate angiogenesis by synthesizing growth factors and components of the basal lamina (Paula Dore-Duffy, 2008; Paula Dore-Duffy & Cleary, 2011; Hirschi & Damore, 1996). Consequently, pericytes may be key regulators of repair after HI-related brain injury in the neonatal brain and offer an opportunity as a potential therapeutic target.

### 2.4 | Astrocytes

Astrocytes are the most abundant class of cells in the NVU (N. J. Abbott, 2002). They cover BECs and pericytes, and approximately 98% of the cerebral vascular surface area with their extensions termed astrocytic end foot processes (Mathiisen, Lehre, Danbolt, & Ottersen, 2010). Their unique form allows them to create an interface between BECs and neurons. Consequently, they provide nutritive support for neurons and regulate endothelia vascular permeability. Therefore, pericytes and astrocytes contribute to the establishment of the specialized phenotype of the BBB (Kimelberg & Nedergaard, 2010). This theory is supported by numerous *in vitro* studies demonstrating that co-cultures of BECs with astrocytes generate much tighter barrier properties than BECs cultures without astrocytes (Helms et al., 2016).

HI injury in the neonatal brain is associated with extensive astrogliosis (Chavez-Valdez, Martin, Flock, & Northington, 2012; C. Y. Chen et al., 2015; Huang et al., 2017; Qiu et al., 2007; Sullivan, Bjorkman, Miller, Colditz, & Pow, 2010; Teo, Morris, & Jones, 2015; Virgintino et al., 2014). Astrocytes exhibit morphologic changes (hypertrophy with swelling of end-feet) immediately after exposure to a HI-related insult. They proliferate and glial fibrillary acidic protein (GFAP) expression increases resulting in the formation of glial scars in the damaged area associated with HI. The role of reactive astrogliosis remains unclear, but most likely has a dual function in the evolution of HI-related brain lesions, particularly, in neonates. On one hand astrocytes can accentuate brain damage by releasing neurotoxic and inflammatory substances (pro-inflammatory cytokines, iNOS), alternatively, they also release beneficial anti-inflammatory cytokines (Sofroniew, 2015). The relative balance of pro-inflammatory and anti-inflammatory cytokines provided by astrocytes probably depends upon the severity of the insult and duration of recovery. Activated astrocytes also release angiogenic factors such as VEGF, which can decrease TJ protein expression and, consequently, increase BBB permeability after stimulating signaling pathways that bind to the VEGF-R2 receptor on BECs (Argaw et al., 2012; Argaw, Gurfein, Zhang, Zameer, & John, 2009; Dobrogowska, Lossinsky, Tarnawski, & Vorbrodt, 1998).

Aquaporin 4 (AQ4) is a major water channel in the mammalian brain that is highly expressed on the astrocytes end-foot processes (Chu et al., 2016). Increased AQ4 expression in the early hours after HI injury contributes to the development of edema formation and astrocytic swelling (Fukuda & Badaut, 2012). However, the effects of HI on AQ4 expression in the neonatal brain remain controversial. AQP4 expression increases on astrocytic end-feet in the border regions of injured tissues up to 72 h after injury and normalizes within 28 days after ischemic stroke in neonatal rats (Badaut et al., 2007). Expression of AQ4 expression was also increases in conjunction with MRI determined brain edema. Its expression also increased in the ependyma and meninges but decreased in the striatum of piglets exposed to HI (H. W. Wang, Wang, & Guo, 2012).

### 2.5 | Microglia

Perivascular microglia/macrophages are in intimate contact with the BBB, particularly surrounding penetrating vessels and venules, and have a role as the first immune defense against pathogens invading the CNS. Perivascular microglia extend processes that surround brain microvessels and regulate the uptake of some macromolecules (Ransohoff & Perry, 2009). In addition to their role in immune responses, microglia also stimulate angiogenesis via VEGF related mechanisms and secrete multiple trophic factors in developing brain (Mallard, Tremblay, & Vexler, 2018). Microglia are activated within hours after HI-related injury probably because they are the first immune cells to respond to inflammatory signals in the brain (Barrios-Anderson et al., 2019; C. Y. Chen et al., 2015; McRae, Gilland, Bona, & Hagberg, 1995). Microglial morphology changes include increases in the size and ramifications with the development of short thick processes. Their numbers increase and surface proteins and enzyme expression are modified. Activated microglia serve a phagocytic role but also secrete a variety of mediators such as free radicals and pro-inflammatory cytokines that spread inflammatory signals and facilitate recruitment of monocytes/macrophages. Activated microglia also potentiate NVU damage by enhancing MMP-mediated injury (Hagberg, Gressens, & Mallard, 2012; Hagberg et al., 2015; Y. X. Jin, Silverman, & Vannucci, 2009; Lai et al., 2017). They appear as damaging cells after the initial onset of injury. This is consistent with the experimental evidence showing that reduced microglia activation diminishes brain injury (Arvin et al., 2002; Dommergues, Plaisant, Verney, & Gressens, 2003). On the other hand, microglia and other immune cells can also enhance repair in the later phases after injury. Microglial phagocytic activity is crucial to remove cellular debris before tissue remodeling can progress. Complete inhibition of microglial activation has also been shown to result in extensive brain damage after ischemic injury (Faustino et al., 2011; Lalancette-Hebert, Gowing, Simard, Weng, & Kriz, 2007). The mediators controlling the shift from the inflammatory to the repair phase remain to be identified.

**Figure 1:**
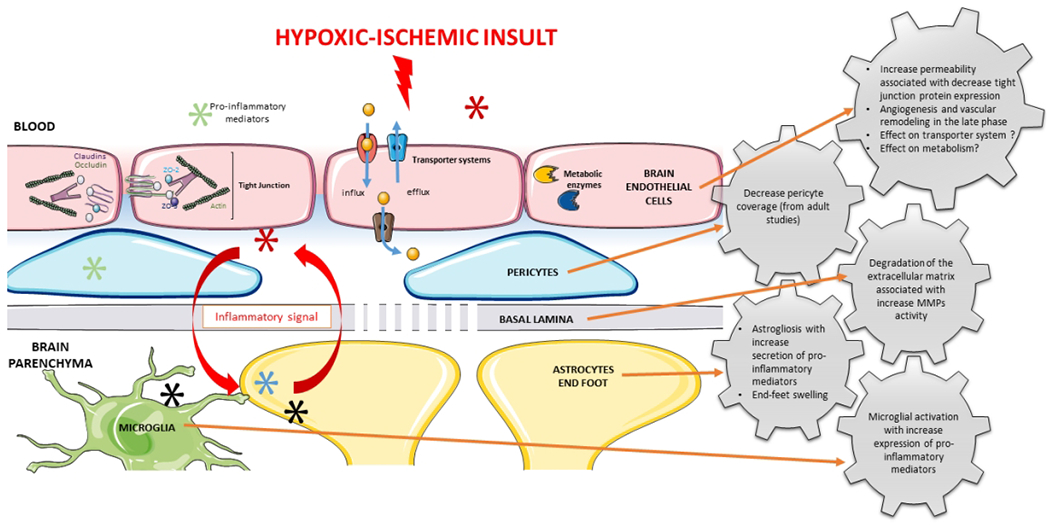
A summary of the neurovascular unit cellular responses after hypoxia ischemia in the neonatal brain

The NVU is a complex multicellular system. Each component of the system is interdependent upon the other constituents and, consequently, the function and regulation of the entire system are dependent upon all of its components. Many variables of the NVU remain to be investigated in the developing brain such as metabolic activities. The most widely reported effects of HI-related insults on the NVU are the increases in the permeability of the BBB. However, the effect of HI injury upon consequences in the later life as well as sex differences remains to be investigated. The effects of HI-related injury upon potential alterations of the NVU in the neonatal brain are graphically summarized in figure 1.

## 3 | Effect of therapeutic interventions in the NVU

### 3.1 | Therapeutic hypothermia

The only strategy approved to treat HIE is therapeutic hypothermia. This strategy is only approved to treat full term newborns and is unfortunately only partially protective primarily after exposure to moderate HIE (Gluckman, Gunn, & Wyatt, 2006; Jacobs et al., 2013; Natarajan, Pappas, & Shankaran, 2016; Shankaran, 2012). Minimal changes in body temperature have been shown to affect the function of the BBB (Kiyatkin & Sharma, 2009). Hyperthermia increases albumin extravagation, activates astrocytes and increases markers of brain edema suggesting that increases in body temperatures predispose to BBB leakage (Kiyatkin & Sharma, 2009). However, severe hypothermia also activates glia, induces cellular abnormalities and increases BBB leakage but reduces brain water content. Nonetheless, there is a paucity of information regarding the effects of temperature on the NVU after exposure to HI-related injury in neonates. Therefore, the available information regarding temperature control and the NVU requires extrapolation from studies *in vitro* and in adult subjects.

Hypothermia initiated soon after ischemia attenuates BBB dysfunction in adult rodents (Baumann, Preston, Slinn, & Stanimirovic, 2009; Preston & Webster, 2004; Zhao et al., 2018). The effects of hypothermia on BBB dysfunction were associated with inhibition of neuroinflammation including decreases in chemokine expression, shifts toward anti-inflammatory microglial phenotypes and reductions in multiple markers of brain injury (Zhao et al., 2018). The effects of *in vitro* oxygen-glucose deprivation on BECs, astrocytes and neurons are also temperature-dependent (Lyden et al., 2018). Hypothermia prevents pericyte separation from the basement membrane and consequent disorganization of the BECs monolayer after ischemic stroke (Duz et al., 2007). Hypothermia also inhibits the loss of the components such as collagen IV and agrin from the basal lamina (Baumann et al., 2009; Hamann et al., 2004; J. E. Lee, Yoon, Moseley, & Yenari, 2005). Therefore, the neuroprotective efficacy of hypothermia to treat HIE in newborn may be a consequence in part of its effects on the NVU based upon the studies *in vitro* and in adult subjects summarized above.

### 3.2 | Erythropoietin

Erythropoietin (EPO) is an attractive anti-inflammatory neuroprotective agent. EPO binds to its receptor expressed on the neuronal membranes, astrocytes and microglial cells to accomplish its anti-inflammatory effects. Numerous studies have shown that EPO attenuates the effects of HI-related injury in neonatal subjects (van der Kooij, Groenendaal, Kavelaars, Heijnen, & van Bel, 2008). However, there is a paucity of information concerning the effects of EPO on the constituents of the NVU in neonates. Treatment with EPO enhances angiogenesis by stimulating the VEGF signaling pathway in neonatal rodents exposed to anoxia (Yan et al., 2016). It also potentially enhances angiogenic responses by increasing CD34+ cells, VEGF and Ang-1 after exposure to HI in neonatal rats (Zhu et al., 2014) and induces neurogenesis and angiogenesis *in vitro* in a co-culture system of endothelial and neural progenitor cells derived from the subventricular zone of adult mice (L. Wang et al., 2008). Pretreatment with EPO reduces brain infarct volume and preserves the BBB integrity after ischemic stroke in adult rodents (Bahcekapili, Uzum, Gokkusu, Kuru, & Ziylan, 2007). Treatment after ischemic injury also preserved BBB integrity by reducing TJ degradation and MMP activity (R. L. Wang et al., 2015). Therefore, some of the neuroprotective effects of EPO could be attributed to their effects on the NVU.

### 3.3 | Other therapeutics

#### 3.3.1 | Stem cell therapy

Stem cell therapy ameliorates neurological deficits after HI-related injury in neonatal subjects (Nabetani, Shintaku, & Hamazaki, 2018). Cell-based therapies derived from umbilical cord blood are rich in endothelial progenitor cells (Pimentel-Coelho, Rosado-de-Castro, da Fonseca, & Mendez-Otero, 2012). Thus, the potential exists that the endothelial cell progenitors have the ability promote neovascularization in tissues damaged by HI. In additon, stem cell therapy has been shown to enhance angiogenesis and reduce damage to the BBB in subjects exposed to HI. Administration of human umbilical vein endothelial cells (HUVECs) attenuates injury to the NVU after exposure to HI (Y. C. Lee, Chang, Wu, & Huang, 2018; Wu et al., 2013). Similarly, treatment with umbilical cord blood CD34+ cells also reduces neuronal damage, decreases astrogliosis and enhances vascular repair after HI (Yu et al., 2018). However, safety profile of stem cell therapy remains to be determine because this therapy could be associated with serious side effects including carcinogenesis and immunosuppression (Titomanlio et al., 2011).

#### 3.3.2. | Anti-cytokines therapy

Targeting pro-inflammatory cytokines could represent a potentially significant therapeutic strategy to treat perinatal HI injury. Recently, we demonstrated encouraging neuroprotective capacities of anti-cytokine monoclonal antibodies (mAbs) after ischemic-reperfusion (I/R) brain injury in the ovine fetus (Disdier, Chen, Kim, Threlkeld, & Stonestreet, 2018). The most important findings were that systemic administration of the anti-IL-1 β mAb attenuated brain injury associated with modulation of the neuro-immune response and improved in l/R-related increases in BBB permeability across multiple brain regions measured with an inert non-specific molecule (X. D. Chen et al., 2015). Similarly, we have also shown that systemic infusions of anti-IL-6 mAb diminished the ischemia-related increases in BBB permeability 24 h hours after ischemic injury, modifying tight junction and plasmalemma vesicle protein expression in the fetal brain (J. Y. Zhang et al., 2015).

#### 3.3.3 | Metformin

Metformin is a widely prescribed drug to treat type 2 diabetes mellitus and metabolic syndrome. It has anti-inflammatory and anti-oxidative effects potentially beneficial after exposure to HI in neonates. In adults, metformin has been shown to promote neurogenesis and protect BBB integrity in experimental stroke (Q. Jin et al., 2014; Takata et al., 2013). Treatment with metformin also attenuates tight junction and protein adherens junction degradation, prevents pericyte loss, reduces the astrocyte and microglia activation and down-regulates neuro-inflammation after HI in neonatal rodents (Fang et al., 2017).

#### 3.3.4 | Other potential therapeutics

Other studies have attempted to attenuate HI related brain injury (Nair & Kumar, 2018). Xenon, N-acetylcysteine and melatonin are free radical scavengers that have demonstrated promising neuroprotective effects in animal models of HI-related brain injury. These agents have demonstrated beneficial effects by reducing microglial activation or astrogliosis. However, studies have not examined the effects of these agents specifically on changes on the elements of the NVU after exposure the HI related injury in the neonatal brain. Single-cell technologies especially single-cell RNA sequencing could help to identify cell-specific variables such as patterns of mRNA and protein expression in the developing brain with respect to the organizational hierarchy and structural diversity of cells in context with brain injury (Fan et al., 2018; Grant et al., 2019; Q. Mu, Chen, & Wang, 2019; Polioudakis et al., 2019). These findings could probably help to identify highly selective pharmacological targets for perinatal astrocytes, microglia and other cellular components of NVU considering the complex interactions among elements of the NVU system.

## 3 | Conclusions

Exposure to HI initiates a wide variety of deleterious effects on the neonatal brain resulting in long-term consequences later in life. The NVU represents an important component in the pathophysiology of neonatal HI-related brain injury. However, many characteristics of the NVU remain to be elucidated after exposure to HI. Although BBB permeability increases immediately after the onset of HI in perinatal brain, there is a paucity of knowledge regarding its effects on transport function and metabolism in BECs. Similarly, studies are still required to understand the impact of HI on other components of NVU as well as their interactions and role of this system during development. Prevention of neurovascular damage or enhancement of repair represents a potentially important therapeutic strategy to attenuate brain injury.

## Figures and Tables

**Table 1:** Summary of studies on the BBB permeability after neonatal HI brain injury

REFERENCES	ANIMAL MODEL	INDICATOR OF INCREASE BBB PERMEABILITY
**Rodent models**		
Svedin, Hagberg, Savman, Zhu, & Mallard, 2007	Mice HI model P9, 50 or 60 min of 10 % O_2_ hypoxia	Extravagation of IgG up to 24 h after HI
**W. Q. Chen, Jadhav, Tang, & Zhang, 2008** W. Q. Chen et al., 2009	Rat HI model P7, 2 h of 8 % O_2_ hypoxia	Extravagation of IgG 24 h after HI
**Ferrari, Nesic, & Perez-Polo, 2010**	Rat HI model P7, 90 min of 8 % O_2_ hypoxia	Increases permeability to fluorescein from 24 h after HI to 7 days, normalization after 21 days
**Tu et al., 2011** **Tu, Lu, Huang, Ho, & Chou, 2012**	Rat HI model P7, 2 h of 8 % O_2_ hypoxia	Extravagation of IgG 24 h after HI
**D. Yang et al., 2013**	Rat HI model P7, LPS-sensitized, 80 min of 10 % O_2_ hypoxia	Increases permeability to fluorescein 24 h after HI
Ek, D’Angelo, Baburamani, et al., 2015	Mouse HI model P9, 50 min of 10 % O_2_ hypoxia	Increases permeability to sucrose, peak at 6 h after HI and normalization in 3 days
**Min et al., 2015**	Rat HI model P7; 2.5 h of 8 % O_2_ hypoxia	Extravagation of IgG 24 h after HI
W. T. Zhang et al., 2016	Rat HI model P7; 2.5 h of 8 % O_2_ hypoxia	Extravagation of cadaverine 4 h after HI and 3kD and 40kD dextrans 24 and 48 h after HI Extravagation of IgG 48 h after HI
Ma et al., 2017	Rat HI model P7, 90 min of 8 % O_2_ hypoxia	Extravagation of IgG 48 h after HI
**Sheep model**		
Sadowska et al., 2012	30 min bilateral carotid occlusion in the fetal sheep (125-129 days of gestation)	Increases permeability to amino-isobutyric acid up to 48 h of reperfusion and with a peak at 4 h
**Yawno et al., 2012**	10 min umbilical cord occlusion in the fetal sheep (130 days of gestation)	Extravagation of albumin after 48 h of reperfusion
Baburamani, Castillo-Melendez, & Walker, 2013	10 min umbilical cord occlusion in the fetal sheep (130 days of gestation)	Extravagation of albumin after 24 h and 48 h of reperfusion
**Castillo-Melendez et al., 2015**	Single uterine artery ligation (105 days of gestation)	Extravagation of albumin 24 h after natural birth

## SIGNIFICANCE

Many brain related injuries can be attributed hypoxia-ischemic events at birth, which result in neurodevelopmental disabilities and place a lifelong burden on parents and society. This event in the developing and immature brain can cause significant mortality and result in long-term neurological abnormalities. Alterations in the components of the neurovasculature represent key contributors to hypoxic-ischemic related brain injury but still are not fully characterized in the neonatal brain. In addition, they also potentially represent promising pharmacological targets. In this review, we summarize the literature that has reported modifications in the neurovascular unit and potential beneficial drug candidates.

## Abbreviations

BBB: Blood-Brain Barrier
CNS: Central Nervous System
BEC: Brain endothelial cell
NVU: Neuro-vascular Unit
ECM: Extracellular Matrix
HI: Hypoxia ischemia
HIE: Hypoxic-ischemic encephalopathy
TJ: Tight junction
SLC: Solute carrier
ABC: ATP binding cassette
VEGF: Vascular endothelial growth factor
FGF-2: fibroblast growth factor 2
MMP: Matrix metalloproteinases
HIF-1α: Hypoxia-inducible factor 1-alpha
GFAP: Glial fibrillary acidic protein
AQ4: Aquaporine 4
EPO: erythropoietin
